# Transumbilical Single-Site Laparoscopic Intraperitoneal Closure of the Internal Inguinal Ring for Pediatric Inguinal Hernia

**DOI:** 10.3389/fped.2022.855537

**Published:** 2022-03-15

**Authors:** Yi Ji, Yanan Li, Xuepeng Zhang, Tong Qiu, Siyuan Chen, Zhicheng Xu

**Affiliations:** ^1^Department of Pediatric Surgery, West China Hospital of Sichuan University, Chengdu, China; ^2^Pediatric Intensive Care Unit, Department of Critical Care Medicine, West China Hospital of Sichuan University, Chengdu, China

**Keywords:** pediatric inguinal hernia, transumbilical laparoscopic herniorrhaphy, internal inguinal ring, follow-up, outcomes

## Abstract

**Background:**

A new novel technique for pediatric inguinal hernia (PIH) repair, namely, transumbilical single-site laparoscopic intraperitoneal closure (TUSLIC) of the internal inguinal ring (IIR) with a single instrument, was introduced. The short-term follow-up of TUSLIC for PIH was compared with that of transabdominal multiple-site laparoscopic extraperitoneal closure (TAMLEC) for PIH.

**Methods:**

Descriptive variables, perioperative clinical features, and short-term outcomes were retrospectively analyzed and compared between the patients who underwent TUSLIC and those who underwent TAMLEC.

**Results:**

In total, 289 patients were enrolled in this study. Of these, 190 patients received TUSLIC, and 99 patients received TAMLEC. The descriptive variables (including sex, age, weight, and preoperative diagnosis of patients) were comparable between the two groups (*P*-values were 0.12, 0.71, 0.69, and 0.23, respectively). The mean operative times for unilateral hernia repair and bilateral hernia repairs in TAMLEC group were significantly less than those in TUSLIC group (*P* < 0.01). The values of surgical site infection, umbilical bleeding, testicular atrophy, iatrogenic ascent of the testis, and secondary hydrocele were not significantly different between the two groups. There were no suture granulomas, and recurrence occurred in TUSLIC group, though at a significantly lower rate than in TAMLEC group (*P* < 0.05).

**Conclusions:**

TUSLIC is a feasible, safe, and reliable minimally invasive method for PIH. Compared with TAMLEC, TUSLIC has the advantages of minimized complications and a low recurrence rate.

## Introduction

The laparoscopic pediatric herniorrhaphy (LPH) operation was first described by Montupet in 1993 using a purse-string suture technique, in which the internal inguinal ring (IIR) was closed with nonabsorbable threads (1, 2). The method of laparoscopic percutaneous extraperitoneal closure of IIR, as introduced by Takehara, is one of the simplest and most reliable operations for pediatric inguinal hernia (PIH) (3). Conventional laparoscopic suturing procedures required three ports. These procedures are technically challenging and were not easily performed even by experienced pediatric surgeons (4).

The procedure of transabdominal multiple-site laparoscopic extraperitoneal closure (TAMLEC) of IIR has been developed for PIH repairs. However, extraperitoneal hernia sac ligation and knot burial subcutaneously in the management of TAMLEC have resulted in some complications, such as stitch sinus, infection, granuloma, and puckering of the skin. In addition, recurrent PIHs may occur due to loose sutures, which slowly cut through muscle tissue, especially in obese children with thick abdominal walls (5, 6). Recently, the percutaneous internal ring suturing (PIRS) requiring only a single umbilical port is used for pediatric inguinal hernia repair (3, 7–10). PIRS is technically easy with a short learning curve (11, 12). PIRS has satisfactory cosmesis and the advantage of identifying the patency of the contralateral processus vaginalis. However, it has been reported that PIRS had relatively higher rates of postoperative complications and recurrence (13). In the present study, we have established a new approach for PIH repair: transumbilical single-site laparoscopic intraperitoneal closure (TUSLIC) of IIR with a single instrument. In this study, we will present this new novel procedure of TUSLIC and analyze our initial experiences.

## Materials and Methods

### Design and Study Population

This was a retrospective study of patients with PIH who underwent TUSLIC and TAMLEC between January 2020 and January 2021. Institutional review board approval for this retrospective case series was obtained at West China Hospital of Sichuan University. The inclusion criteria were as follows: patients age of 0–14 years and presence of clinically confirmed groin hernia (by ultrasonography examination); patients had received either TUSLIC or TAMLEC. The exclusion criteria were: patients with comorbidities, including Hirschsprung's disease (HD), abdominal tumors, and cryptorchidism. Patients' parents were given the option to choose the treatment (either TUSLIC or TAMLEC). The patients' parents or guardians gave written, informed consent.

### Study Outcomes

Descriptive variables, perioperative clinical features, and short-term outcomes were analyzed and compared between the patients with TUSLIC and patients with TAMLEC. The primary outcome of this study was recurrence. Secondary outcomes included intraoperative and postoperative complications, conversions to open surgery, and operative time (ORT). We hypothesized that patients receiving TUSLIC might have less recurrence and postoperative complications than those receiving TAMLEC.

### Surgical Technique

#### The Procedure of TAMLEC

In the TAMLEC procedure, the patient was placed in a supine position with a monitor at the patient's feet. The operator stood on the opposite side of the inguinal hernia, and the camera assistant stood on the other side. A 5 mm incision was made through the center of the umbilicus with the open Hasson technique (14) to establish the pneumoperitoneum at a pressure of 6–8 mmHg with a flow rate of 3–6 L/min. A 5 mm trocar and a 30° laparoscope were introduced into the peritoneal cavity. Then, a second 3 mm incision was made for direct insertion of a 3 mm grasper without a trocar at the intersection point between the anterior midline and the level of 2.0–4.0 cm distal to the umbilicus.

Laparoscopy was started by inspection of the pelvis and bilateral IIRs. A modified Kirschner wire with a single 2–0 non-absorbable thread was introduced vertically through a 2 mm eyelet at the surface projection of IIR to the preperitoneal space, in which the ilioinguinal nerve, as well as penetration of the peritoneum, were avoided. With the help of a 3 mm grasper traction on the peritoneum, the Kirschner wire easily traversed the epigastric vein and vas deferens in males beneath the peritoneum along the medial and inferior border of the IIR. The peritoneum was pierced medially by the wire, and the loop end of the thread was left intraperitoneally with the other end outside the abdomen when the wire was pulled out of body. Subsequently, the wire with another single 2–0 non-absorbable thread was inserted through the previous eyelet again, guided under the peritoneum of the superior and lateral border of the IIR, advanced over the spermatic cord vessels, and then pierced into the peritoneum where the loop end of the suture was left before. The end of the loop thread was placed through the loop at the tip of the Kirschner wire using a 3 mm grasper, after which the end of the double threads was pulled out by withdrawing the wire, the hernia sac was highly ligated extraperitoneally by tying both corresponding threads tightly, and the knots were buried subcutaneously.

If a contralateral potent processus vaginalis (PPV) was present, the TAMLEC procedure was repeated immediately without additional trocars and incisions. Finally, all the instruments were removed, the abdomen was desufflated, and the incisions (three in unilateral repair, four in bilateral repairs) over the abdominal wall were closed and covered with adhesive paper strips. In patients with hydrocele, the hydrocele was punctured from the scrotum.

#### The Procedure of TUSLIC

**Figure 1:**
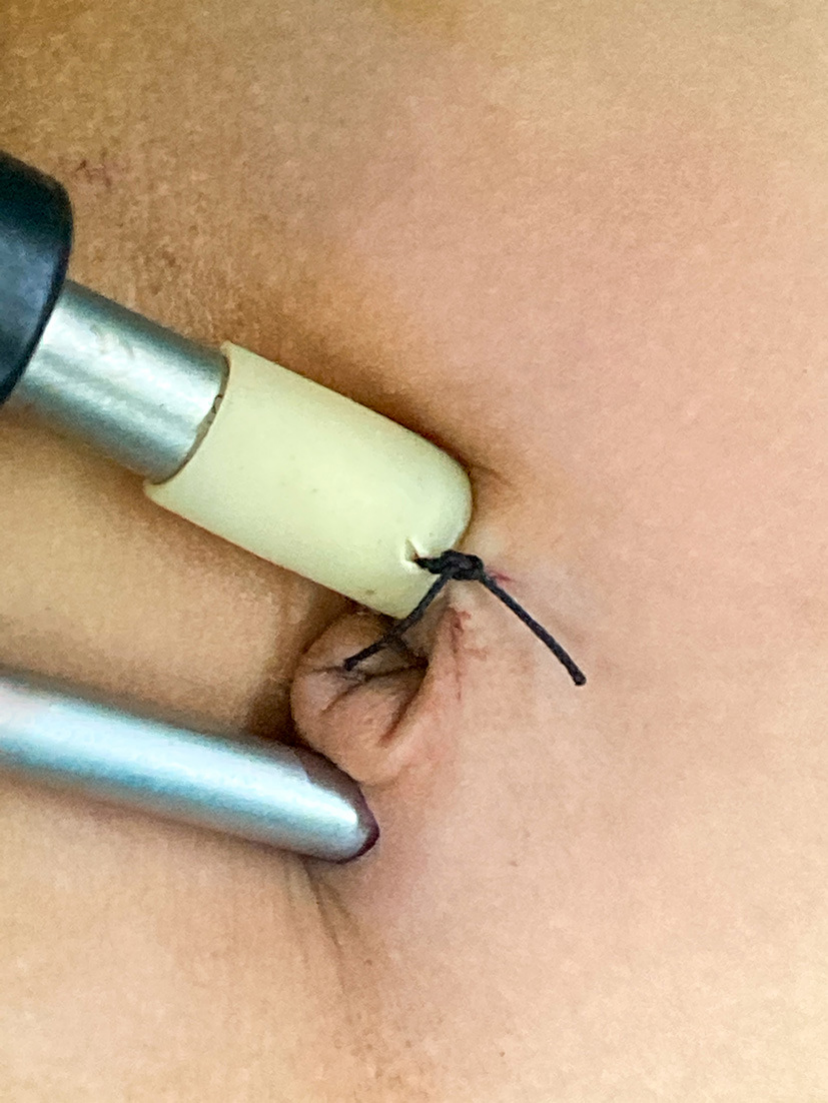
A 5 mm trocar and a 30° laparoscope were introduced into the peritoneal cavity through the left rim of the umbilical ring with the open Hasson technique. Another 3.0 mm incision was made for placement of the 3 mm laparoscopic needle holder or scissors at the right rim of the umbilical ring without a trocar.

In the TUSLIC procedure, the patient was placed in a supine position with a monitor at the patient's feet. The surgeon stood on the head side of the patient. A 5 mm incision was made through the left rim of the umbilical ring with the open Hasson technique to establish pneumoperitoneum at a pressure of 6–8 mmHg with a flow rate of 3–6 L/min (Figure 1). A 5 mm trocar and a 30° laparoscope were introduced into the peritoneal cavity. A second 3 mm incision was made for placement of the working instrument at the right rim of the umbilical ring without a trocar.

Laparoscopy was started by inspection of the pelvis and both IIRs. An 18-G vascular catheter was pierced into the preperitoneal space between the peritoneum and the structures of the vas deferens. The spermatic cord in males was expanded with 1–5 ml saline solution. The needle with double 2–0 braided polyester threads punctured the body surface from the outside at the right or left lower quadrant into the peritoneal cavity, 2 cm above and lateral to the IIR, leaving one end of the double threads outside the abdominal wall. Under direct vision, the needle was driven to pierce the peritoneum at 5 o'clock for the beginning point of suture, advanced carefully in front of the spermatic cord vessels and vas deferens, beneath the peritoneum along the inferior margin of IIR, and pulled out of the peritoneum at 7 o'clock. Then, the needle was manipulated headward and reinserted into the preperitoneal space at 7 o'clock, guided along the medial, superior, and lateral margins of the IIR, passed over the inferior epigastric vein, and then drawn from the previous peritoneum hole at 5 o'clock. If the opening of the IIR was large, more steps were carried out to ensure the uninterrupted circle seam surrounding the IIR.

**Figure 2:**
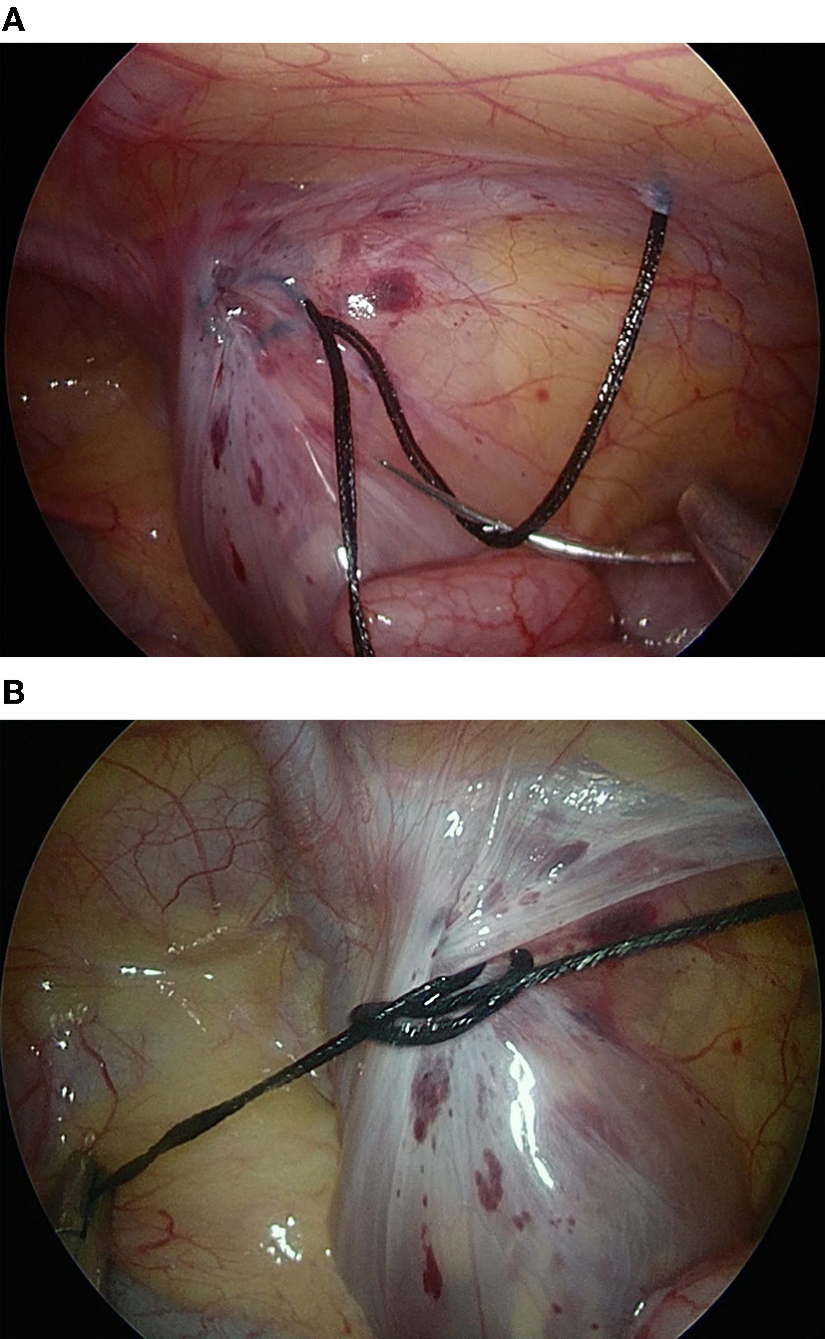
The tip of the short end of the threads was grasped by a needle holder and rotated 360° either under or above the long limb of the thread, forming a loop that was passed through the first loop **(A)**. The loop was pushed in a downward and medial direction to the pelvic cavity, while the long end of the double threads outside the body was pulled upward **(B)**.

Once the circular seam around the IIR was completely established without any skip areas, the long end of the double threads was held outside of the abdomen by the surgeon's left hand. The tip of the short end of the double threads was grasped and rotated 360 degrees either under or above the long limb of the double threads, forming a loop (Figure 2A), which was passed through that loop and circulated twice around the long limb to make a surgeon's knot. The short end of the double threads was pushed in a downward and medial direction to the pelvic cavity, while the long end of the double threads was pulled upward (Figure 2B), which was repeated as above 2–3 times to form a locking knot. All threads were cut off, and a 5 mm long stump of the knot was left.

**Figure 3:**
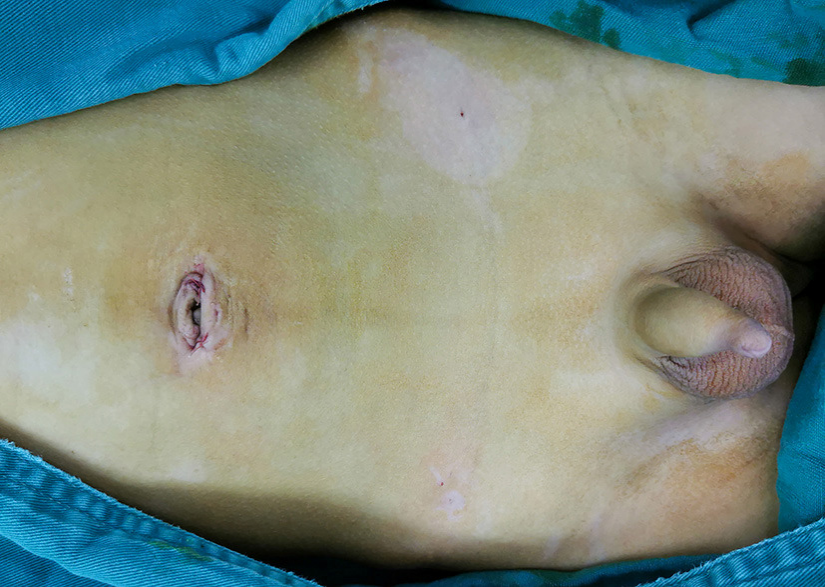
The 5 and 3 mm incisions on the bilateral rims of the umbilical ring were closed. The puncture holes in the abdominal wall that were formed by the needle were left open without a dressing.

If a contralateral PPV was present, the TUSLIC procedure was performed simultaneously without additional trocars or incisions. Finally, all the instruments were removed, and the incisions at the bilateral rim of the umbilical ring were closed (Figure 3) and covered with adhesive paper strips. The puncture holes on the abdominal wall in the right or left lower quadrant were left open without dressing. In patients with hydrocele, the hydrocele was punctured from the scrotum.

### Data Collection and Statistical Analysis

Patients were analyzed for their descriptive variables and perioperative clinical features (age at operation, sex, body weight, preoperative diagnosis, ORT, unilateral: bilateral repairs, contralateral PPV, and conversion) by reviewing their medical charts. The follow-up data, including the level of surgical site infection (SSI), umbilical bleeding, testicular atrophy, iatrogenic ascent of the testis, secondary hydrocele, suture granuloma, and recurrences, were collected at the last visit to our outpatient clinic according to the medical files.

Independent sample Student's *t*-tests (or Mann–Whitney *U*-test) and chi-squared tests (or Fisher's exact test) were used to compare continuous and categorical descriptive variables, respectively. The results are expressed as the mean with standard deviation (SD) or median with interquartile range. The software applied for statistical calculation was IBM SPSS 22.0 for Windows 10.0 (IBM Corp.). A *P* of < 0.05 was considered statistically significant.

## Results

### Data on Descriptive Variables and Perioperative Clinical Features

There were 299 patients with PIH under 14 years of age who underwent TUSLIC and TAMLEC between January 2020 and January 2021. Among them, 10 patients were excluded from the study because of combined comorbidities (four patients had HD, three patients had retroperitoneal lymphangioma, two patients had oval cystic teratoma, and one patient had cryptorchidism). The remaining 289 patients were enrolled in this study. Of them, 190 patients underwent TUSLIC, and 99 patients underwent TAMLEC. Among 190 patients in TUSLIC group, there were 168 males and 22 females with a mean age of 30.5 ± 24.6 months. Among 99 patients in TAMLEC group, there were 81 males and 18 females with a mean age of 29.4 ± 21.8 months. The descriptive variables, including sex, age, body weight, and preoperative diagnosis, between the two groups were comparable (*P* > 0.05; Table 1).

**Table 1:** Data of the descriptive variables and perioperative features of the two groups.

**Patients (*N*)**	**TUSLIC group (190)**	**TAMLEC group (99)**	***P***
Sex (male/*N*)	0.88	0.82	0.12^†^
**Age (months)**
Mean (SD)	30.5 ± 24.6	29.4 ± 21.8	0.71*^‣^*
Median (IQR)	26.3 (19.2–35.6)	26.1 (18.4–33.9)	
**Body weight (kg)**
Mean (SD)	16.7 ± 8.4	15.8 ± 13.1	0.69*^‣^*
Median (IQR)	13.6 (11.5–17.0)	12.9 (11.2–16.6)	
**Preoperative diagnosis**
Left	59	24	0.23^†^
Right	108	59	0.65^†^
Bilateral	23	16	0.34^†^
Unilateral:bilateral repairs	92:88	55:44	0.25^†^
ORT (unilateral) (min)	18.8 ± 4.8	12.2 ± 2.1	<0.01^‡^
ORT (bilateral) (min)	29.9 ± 5.6	21.5 ± 2.6	<0.01^‡^
Contralateral PPV (*N*)	65	28	0.31^‡^
Conversion (*N*)	0	0	1.0^§^

*TUSLIC, transumbilical single-site laparoscopic intraperitoneal closure; TAMLEC, transabdominal multiple-site laparoscopic extraperitoneal closure; ORT, operative time; PPV, patent processus vaginalis; min, minutes; SD, standard deviation; IQR, interquartile range*.

† *P-value was calculated using the chi-square test*.

# *P-value was calculated using the Mann–Whitney U-test*.

*P-value was calculated using an independent sample Student's t-test*.

§ *P-value was calculated with Fisher's exact test*.

During the operation, all patients performed well in both groups. No patient needed to convert to conventional herniorrhaphy. The value of contralateral PPV was 34.2% in TUSLIC group, which was not significantly different from the 28.3% in TAMLEC group (*P* = 0.31). The ORTs for unilateral hernia repair and bilateral hernia repair in TAMLEC group were significantly lower than those in TUSLIC group (*P* < 0.01). No intraoperative complications occurred in the two groups.

### Short-Term Follow-Up Results

The response rate for the telephone questionnaire and/or clinic interview was 94.8%, including 190 patients with TUSLIC (93.7%) and 99 patients with TAMLEC (97.0%). The data of 15 patients (TUSLIC 12, TAMLEC 3) were not collected. They had incorrect phone numbers, and no family member was contactable for the telephone or clinic interview.

The follow-up time was 9.8 ± 3.4 months in TUSLIC group and 9.6 ± 3.2 months in TAMLEC group (*P* = 0.81). Postoperative complications, including SSI, umbilical bleeding, testicular atrophy, iatrogenic ascent of the testis, and secondary hydrocele, were not significantly different between the two groups (*P* > 0.05; Table 2). Compared with TAMLEC group, there was no suture granuloma and recurrent PIH occurred in TUSLIC group (*P* = 0.01 and 0.04, respectively). In TAMLEC group, one obese patient and two patients with postoperative suture granuloma had inguinal hernias that recurred.

**Table 2:** Short-term follow-up data compared between the two groups.

**Patients (*N*)**	**TUSLIC group (190)**	**TAMLEC group (99)**	***P***
SSI (*N*)	2	0	0.31^†^
Umbilical bleeding	3	0	0.21^†^
Testicular atrophy (*N*)	0	0	N/A
Iatrogenic ascent of the testis	0	0	N/A
Secondary hydrocele (*N*)	0	1	0.17^†^
Suture granuloma (*N*)	0	4	0.01^†^
Recurrence (*N*)	0	3	0.04^†^
Follow-up (m)	9.8 ± 3.4	9.6 ± 3.2	0.81^‡^

*TUSLIC, transumbilical single-site laparoscopic intraperitoneal closure; TAMLEC, transabdominal multiple-site laparoscopic extraperitoneal closure; SSI, surgical site infection; m, month; N/A, data not available*.

† *P-value was calculated with Fisher's exact test*.

*P-value was calculated using an independent sample Student's t-test*.

## Discussion

Transumbilical two-port laparoscopic intraperitoneal closure, which is a well-developed minimally invasive surgery for PIH, leaves almost invisible scars on the abdominal wall and avoids the disadvantages of extraperitoneal closure of IIR (15–18). However, it has not achieved wide acceptance because of its demanding techniques and difficult learning curve (19). In response to these challenges, the TUSLIC of IIR with a single instrument was established in January 2020, which is an improved transumbilical two-port laparoscopic intraperitoneal closure. In the present study, we provided evidence that TUSLIC is a safe and effective procedure for IIR in pediatric population. In addition, the results of our study revealed that TUSLIC had the advantages of minimized postoperative complications and a low recurrence rate comparing to those of TAMLEC. The peri- and postoperative complications, such as SSI, umbilical bleeding, testicular atrophy, iatrogenic ascent of the testis and secondary hydrocele after TUSLIC, were not significantly different from those after TAMLEC. However, the ORT during TUSLIC was significantly longer than that during TAMLEC due to the complex techniques in TUSLIC of IIR with the single instrument.

Recurrence rate is one of the most important outcome measures in PIH operation. It has been demonstrated that recurrence rates after open PIH repair and standard 3 port laparoscopic hernia repair ranged from 0.5 to 4% and 0.7 to 4.5%, respectively (8, 13, 20). In the present study, no cases of recurrence were recorded in patients receiving TUSLIC after a mean follow-up of 9.8 months.

Enlargement of the preperitoneal space with saline solution between the peritoneum along the inferior border of the IIR and the structures of the spermatic cord vessels and vas deferens is a prerequisite for successful TUSLIC. Moreover, normal saline for preperitoneal hydrodissection could predispose patients to the formation of preperitoneal local adhesions and fibrosis (21). It has been demonstrated that peritoneal trauma prior to repair could decrease the possibility of recurrence (3). Therefore, during passing of the threads, the saline liquid in the preperitoneal space may cause more tissue trauma, further promote the formation of healing around the IIR and reduce later recurrences.

Avoidance of damaging vas deferens and spermatic blood vessels is also a major concern during PIH operation. In TUSLIC, a volume of 1–5 ml of saline injection is needed for most patients. For children with a large opening of the hernia sac, a 5–10 ml or greater volume of saline injection could help unfold the redundant peritoneum along the inferior border of the IIR, which is convenient for seaming without a jumping zone and protects the spermatic cord vessels and vas deferens from damage. In the present study, no injury of epigastric or iliac blood vessels occurred in TUSLIC. These complications have been commonly reported in patients receiving PIRS (3, 10). Importantly, it has been revealed that more experienced pediatric surgeon had a lover incidence of these intraoperative complications (7, 12). However, questions still exist as whether TUSLIC, PIRS, or any other PIH repair technique may damage the spermatic cord. In this regard, testicular atrophy has been observed in patients who receiving open inguinal hernia repair. Remarkably, no case of testicular atrophy was recorded in a study of 188 patients receiving PIRS, with a median follow-up of 46 months (7).

During TUSLIC, it is important to note that when the working instrument repeatedly passes the incision at the right edge of the umbilicus, a false path into the peritoneal cavity may be formed, leading to more tissue trauma. This may have been responsible for the two cases of SSI and three cases of umbilical bleeding after TUSLIC. However, these complications were observed to decrease as we gained experience in TUSLIC. For small infants with a small abdominal space, the supine position is tilted 15° with the head down, the emptied bladder and the appropriately raised pneumoperitoneum, all of which can contribute a satisfactory working space. Similarly, it has been reported that complications significantly decreased after 10–25 or 30–35 patients in PIRS (7, 9).

Previous studies confirmed that there was a learning curve for intraoperative complications that reached the benchmarks after each pediatric surgeon performed at least 30–35 cases (11, 12).

The basic technique in TUSLIC is to control the needle tip direction with a single needle holder, which can be practiced by changing the 2-dimensional gripping angle (2-DGA) between the axis of the needle body and needle holder. The 2-DGA can be altered by adjusting the needle end when the needle tip is anchored by the sutured tissue or by rotating the needle body when the needle end is suspended by attached threads from the outside abdominal wall. However, the learning curve of TUSLIC may be long and steep, which requires much simulator training, even for a senior surgeon. Therefore, this may explain why the ORT in patients receiving TUSLIC was much longer than that of patients receiving TAMLEC or PIRS (20, 22–24).

Our study cohort included patients who aged 0–14 years. A subgroup of our patients were adolescents, with an age of 10–14 years. Form the pathophysiology of view, inguinal hernias in adolescent are more similar to that in children than in adults. We could not analyze clinical outcomes of the adolescent subgroup in our current study due to a relatively small sample size. However, no severe complications and recurrence were observed in our adolescent subgroup. In a recent study, the authors reported no postoperative complications and recurrences occurred in a group of 51 adolescents who receiving PIRS with an average follow-up of 44 months (25). These findings, together with our observation, further support that laparoscopic high ligation is a reliable procedure for inguinal hernia repair in adolescent patients.

It is important to consider the limitations of this study. First, this is a retrospective study conducted in a single institution and may not reflect all institutions. A multicenter randomized trial comparing the two treatment modalities is necessary. Second, the follow-up data for a small number of patients enrolled in this research were missing, which may have affected the final statistical results. Third, our study had a relative short follow-up period (mean about 9.5 months). This may be too short to draw some serious conclusions. A more convincing conclusion may require a longer follow-up in the future.

## Conclusion

In conclusion, TUSLIC for PIH is a feasible, safe and reliable minimally invasive procedure for a well-trained surgeon. Compared with TAMLEC, TUSLIC has the advantages of a lower recurrence rate and fewer complications. The TUSLIC procedure for IIR with a single instrument may be considered an alternative option for PIH.

## Data Availability Statement

The raw data supporting the conclusions of this article will be made available by the authors, without undue reservation.

## Ethics Statement

The studies involving human participants were reviewed and approved by the Ethics Committee of West China Hospital (No. 2019-006). Written informed consent to participate in this study was provided by the participants' legal guardian/next of kin.

## Author Contributions

ZX designed the study. ZX and YJ collected data and managed its quality. ZX, YJ, YL, XZ, and TQ performed the statistical analysis and drafted the manuscript. SC and YJ contributed substantially to its revision. All authors read the manuscript carefully, approved the final version, and participated data interpretation.

## Funding

This work was supported by the National Natural Science Foundation of China (grant numbers 81400862 and 81401606), the Key Project in the Science and Technology Program of Sichuan Province (grant number 2019YFS0322), the Science Foundation for The Excellent Youth Scholars of Sichuan University (grant number 2015SU04A15), and the 1·3·5 Project for Disciplines of Excellence Clinical Research Incubation Project, West China Hospital of Sichuan University (grant numbers 2019HXFH056, 2020HXFH048, and ZYJC21060).

## Conflict of Interest

The authors declare that the research was conducted in the absence of any commercial or financial relationships that could be construed as a potential conflict of interest.

## Publisher's Note

All claims expressed in this article are solely those of the authors and do not necessarily represent those of their affiliated organizations, or those of the publisher, the editors and the reviewers. Any product that may be evaluated in this article, or claim that may be made by its manufacturer, is not guaranteed or endorsed by the publisher.

## Abbreviations

PIH: pediatric inguinal hernia
TUSLIC: transumbilical single-site laparoscopic intraperitoneal closure
IIR: internal inguinal ring
TAMLEC: transabdominal multiple-site laparoscopic extraperitoneal closure
LPH: laparoscopic pediatric herniorrhaphy
HD: Hirschsprung's disease
PPV: potent processus vaginalis
ORT: operative time
SSI: surgical site infection
SD: standard deviation
2-DGA: 2-dimensional gripping angle.
